# Lower body mass index potentiates the association between late-night dinner and the prevalence of proteinuria

**DOI:** 10.3389/fendo.2025.1683354

**Published:** 2025-11-10

**Authors:** Jun Muratsu, Hiroshi Akasaka, Kei Kamide, Makiko Morita, Masato Hayashi, Ayaka Nariyama, Kota Okamoto, Tatsuya Wada, Katsuhiko Sakaguchi, Yan Zhang, Daisaku Masuda, Takashi Shigematsu, Koichi Yamamoto, Hiromi Rakugi

**Affiliations:** 1 Department of Nephrology, Rinku General Medical Center, Izumisano, Japan; 2 Department of Geriatric and General Medicine, Osaka University Graduate School of Medicine, Suita, Japan; 3 Department of Hygiene and Preventive Medicine, Iwate Medical University School of Medicine, Yahaba, Japan; 4 Division of Health Sciences, Osaka University Graduate School of Medicine, Osaka, Japan; 5 Department of Nephrology and Hypertension, Sumitomo Hospital, Osaka, Japan; 6 Department of Health Care Center, Rinku General Medical Center, Izumisano, Japan; 7 Department of Cardiology, Rinku General Medical Center, Izumisano, Japan; 8 Osaka Rosai Hospital, Sakai, Japan

**Keywords:** late-night dinner, proteinuria, body mass index, health checkup, microalbuminuria

## Abstract

**Background:**

The presence of proteinuria or microalbuminuria is significantly associated with an increased risk of cardiovascular disease and all-cause mortality. Several studies reported the association between unhealthy eating patterns and proteinuria. While unhealthy eating patterns are a risk factor for obesity, they have also been reported to be a health risk in non-obese people without kidney disease. This cross-sectional study aimed to assess the association between late-night dinner and the prevalence of proteinuria in non-obese subjects with normal renal function.

**Methods:**

The present study included 2,127 participants (1,028 males and 1,099 females) with an estimated glomerular filtration rate ≥ 60 mL/min/1.73 m^2^ and no history of kidney disease who underwent a health checkup at Rinku General Medical Center. To evaluate the impact of late-night dinner on prevalence of proteinuria (defined as dipstick proteinuria of ≥ ±), we applied logistic regression models adjusted for clinically relevant factors.

**Results:**

Late-night dinner was reported in 297 males (28.9%) and 176 females (16.0%). Multivariable adjusted logistic regression models showed that late-night dinner was significantly associated with the prevalence of proteinuria in males. This association remained significant in lower body mass index (BMI) males (BMI< 24.9 kg/m^2^), even after adjusting for clinically relevant factors (adjusted odds ratios were 3.57 [1.34-9.48] and 3.15 [1.22-8.13], respectively). In contrast, this association was not evident in participants with a higher BMI ≥ 24.9 kg/m^2^.

**Conclusion:**

The effect of late-night dinner on proteinuria may vary depending on BMI, particularly in males.

## Introduction

Proteinuria and microalbuminuria are closely associated with the risk of cardiovascular events (1, 2). Microalbuminuria is known to develop in association with conditions such as diabetes and hypertension and has been suggested to promote the progression of atherosclerosis (3).

Previous studies have demonstrated an association between proteinuria and various unhealthy lifestyle behaviors (4–8). Among various unhealthy life behaviors, the relationship between late-night dinner and proteinuria was reported (9, 10). A cross-sectional study of 60,800 participants revealed that habitual skipping breakfast concomitant with late-night dinner was significantly associated with metabolic syndrome and proteinuria, even after adjusting for relevant confounders (10). A retrospective cohort study including 26,764 Japanese from the general population aged ≥ 40 years (mean age was 68 years) showed that late-night dinner was associated with higher risks for proteinuria onset (11). A recent cohort study of 128,594 participants showed that late-night dinner was associated with an increased risk of incident type 2 diabetes in non-obese populations (12).

While proteinuria is commonly prevalent in obese individuals, a previous report found that the association between the prevalence of proteinuria and body mass index (BMI) was observed in the higher and lower BMI groups (13). In addition, we previously reported that the association between skipping breakfast and the prevalence of proteinuria was potentiated in non-obese participants (4).

This cross-sectional study aimed to assess the clinical impact of BMI on the association between late-night dinner and the prevalence of proteinuria in 2,127 participants with normal renal function.

## Methods

### Study population

**Figure 1:**
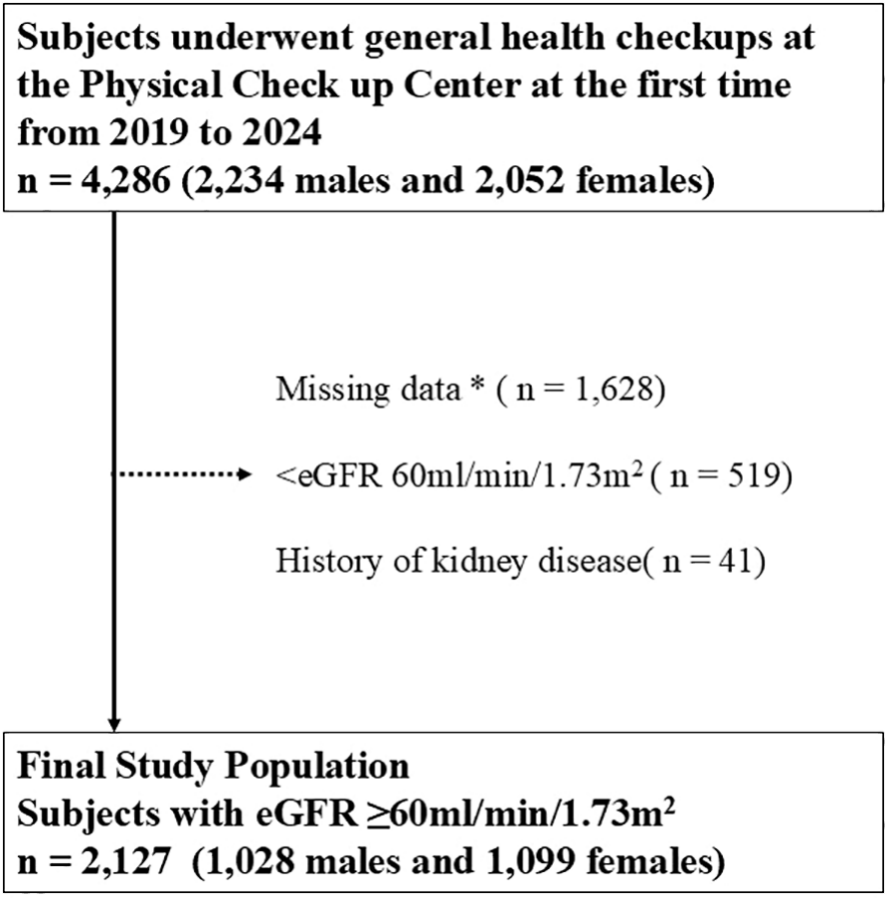
Inclusion and exclusion processes of the present study. * Including age, sex, body mass index, blood pressure, hemoglobin A1c, cholesterol, hemoglobin, aspartate transaminase, alanine aminotransferase, albumin, total cholesterol, triglyceride, high-density lipoprotein cholesterol, low-density lipoprotein cholesterol, fasting blood sugar level, insulin, uric acid, serum creatinine, eGFR, smoking status, drinking frequency, sleeping satisfaction, exercise frequency, presence or absence of late night dinner, and medical histories of diabetes, dyslipidemia, hypertension, hyperuricemia, stroke, and coronary disease. eGFR, estimated glomerular filtration rate.

Eligible participants were 4,286 participants from the general population who underwent a health checkup at the Physical Checkup Center of Rinku General Medical Center between October 2019 and April 2024. The health checkup program aims to facilitate the early detection of disease. Exclusion criteria: not completing the questionnaire and missing data (n = 1,628), eGFR < 60 mL/min/1.73 m^2^ (n = 519), and a history of kidney disease (n = 41). This study included 2,127 participants (1,028 males and 1,099 females) with normal renal function (eGFR ≥ 60 mL/min/1.73 m^2^), no history of kidney disease, and no missing data (**Figure 1**).

### Measurements

Baseline demographic, physical, and laboratory data at the first visit included age, sex, body mass index (BMI = weight [kg]/height^2^ [m^2^]), waist circumference, blood pressure, hemoglobin A1c (HbA1c), hemoglobin, aspartate transaminase, alanine aminotransferase, albumin, total cholesterol, triglyceride, high-density lipoprotein cholesterol, low-density lipoprotein cholesterol, fasting blood sugar level, uric acid, serum creatinine, and eGFR.

Baseline life behaviors for late-night dinner, smoking, heavy alcohol intake, lack of exercise habits, and sleep insufficient, as well as medical histories for hypertension, diabetes mellitus, dyslipidemia, and cardiovascular disease, were evaluated using standardized self-administered questionnaires and interviews by doctors at the participants’ baseline visit. Sleep satisfaction was categorized into satisfaction and insatisfaction. Exercise habits were categorized into over 2 days/weeks and under 2 days/weeks. Daily alcohol consumption was classified into four categories: over 60 g, 40–60 g, 20–40 g, and 0–20 g of ethanol. One standard drink was defined as containing approximately 20 g of ethanol, equivalent to 500 mL of beer, 180 mL of Japanese sake (a traditional Japanese alcoholic beverage), 80 mL of shochu (a Japanese liquor), 60 mL of whiskey, or 240 mL of wine (14). Smoking status was categorized into three groups: current smoking, past smoking, and never. The participants who took dinner within 2 hours before bedtime over 3 days/week were defined as the late-night dinner group.

Laboratory data were measured after overnight fasting. To calculate eGFR, the Japanese formula was used (eGFR [mL/min/1.73 m^2^] = 194 × serum creatinine [mg/dL] − 1.094 × Age [years] − 0.287 × 0.739 [if female]) (15).

The outcome measure of interest was proteinuria (dipstick urinary protein ≥ ±). The results of the urine dipstick tests of proteinuria using Uropaper αIII(EIKEN CHEMICAL, Tokyo Japan) were measured using US-3500 (EIKEN CHEMICAL, Tokyo Japan) and recorded as negative, ±, 1+, 2+, or 3 +. In US-3500, a qualitative urine analysis was performed using the color reaction of test paper from reflection photometry. Waist circumference was measured at the navel level in a relaxed standing position. The tests were conducted under the following conditions: the last food was consumed 14 hours or more and fluids 3 hours or more before the health checkup.

### Statistical analysis

Baseline characteristics between late-night dinner and taking dinner more than 2 hours before bedtime were compared using appropriate statistical tests, including ANOVA, the χ² test, the t-test, the Kruskal–Wallis test, or the Wilcoxon rank-sum test, as applicable.

The association between late-night dinner and prevalence of proteinuria (dipstick proteinuria of ≥ ±) was assessed using multivariable logistic regression models adjusting for the baseline variables, including age, sex, waist circumference, smoking status (current smoking, past smoking, or never), drinking frequency (ethanol amount: over 60 g, 40–60 g, 20–40 g, or 0–20 g), sleeping satisfaction (satisfaction or insatisfaction), presence or absence of late-night dinner, and medical history of diabetes, dyslipidemia, hypertension, hyperuricemia or cardiovascular disease. Medical history of diabetes mellitus was defined as a fasting blood glucose level of 126 mg/dL or higher, an HbA1c level of 6.5% or higher, or the use of diabetes medication.

Proteinuria and microalbuminuria are closely associated with the risk of cardiovascular events (1, 2). Trace proteinuria, which is usually defined as ± by dipstick urinalysis, showed over microalbuminuria and is considered equivalent to microalbuminuria (16). Trace proteinuria is associated with metabolic syndrome, hypertension, and diabetes (17). Some reports suggested that trace proteinuria was also associated with cancer incidence (18). It has been reported that the rate of medical checkups is related to the incidence of end-stage renal failure, and it is important to educate people about the importance of medical checkups (19). Since one of the purposes of this article presentation is to raise health awareness, proteinuria ± or higher was defined as proteinuria from the perspective of preventive medicine.

In previous reports, the prevalence of proteinuria showed a J-shaped relationship with BMI in a cross-sectional study (13). To clarify the effect of BMI on the relationship between late-night dinner and the prevalence of proteinuria, each sex was divided into thirds by BMI. The association of late-night dinner with prevalence of proteinuria was assessed in three subgroups with BMI (males: < 22.3, 22.3-24.9, and ≥ 24.9 kg/m^2^; females: < 20.3, 20.3-23.0, and ≥ 23.0 kg/m^2^, respectively) and waist circumference (males <83.0, 83.0–90.1, and ≥90.1; females <75.0, 75.0–83.5, and 83.5 cm, respectively).

Categorical variables were expressed as numbers (percentages), and continuous variables were shown as mean ± standard deviation or median (interquartile range), as appropriate. Statistical significance was set at *P* < 0.05 if not specified. All statistical analyses were performed using Stata, version 14.2 (Stata Corp., http://www.stata.com).

### Ethics approval

This study was approved by the human ethics committees of Rinku General Medical Center and was conducted according to the principles of the Declaration of Helsinki (approval No. 2024-040). Written informed consent was obtained from all participants to provide medical information and blood samples before the checkup examinations, and each participant had the right to refuse the use of their results. We accessed these data from 2025/02/20. We could not access to information that could identify individual participants after data collection.

## Results

The study population consisted of 1,028 males (mean age 55 ± 14 years) and 1,099 females (mean age 54 ± 14 years) stratified by BMI levels (**Tables 1A**, **1B**). In males, 88 (24.9%), 85 (25.6%), and 124 (36.3%) took late-night dinner with BMI < 22.3, 22.3 ≤ BMI < 24.9, and 24.9 ≤ BMI, respectively. In females, 51 (13.3%), 54 (15.8%), and 71 (18.9%) took late-night dinner with BMI < 20.3, 20.3 ≤ BMI < 23.0, and 23.0 ≤ BMI, respectively. In males, 22 (6.3%), 22 (6.6%), and 26 (7.6%) showed proteinuria above ± in BMI < 22.3, 22.3 ≤ BMI < 24.9, and 24.9 ≤ BMI, respectively. In females, 15 (3.9%), 6 (1.8%), and 17 (4.5%) showed proteinuria above ± in BMI < 20.3, 20.3 ≤ BMI < 23.0, and 23.0 ≤ BMI, respectively.

**Table 1A:** Clinical characteristics of 1,028 males stratified by body mass index (BMI) levels.

Parameters	BMI<22.3 n= 354 (32.4%)	22.3 ≤BMI <24.9 n= 332 (30.4%)	24.9 ≤BMI n= 342 (31.3%)	*P* value
Age (years)	55 ± 14	57 ± 14	53 ± 13	0.060
Height (cm)	170.1 ± 5.9	169.6 ± 6.2	170.2 ± 6.3	0.539
Weight (kg)	59.4 ± 5.8	67.8 ± 5.4	80.2 ± 9.6	<0.001
BMI (kg/m^2^)	20.5 ± 1.4	23.5 ± 0.8	27.6 ± 2.5	<0.001
Waist circumference (cm)	78.5 ± 5.8	86.8 ± 4.7	96.0 ± 7.1	<0.001
Medical history, n (%)				
Hypertension	62 (17.5)	88 (26.5)	120 (35.1)	<0.001
Diabetes mellitus	30 (8.5)	32 (9.6)	46 (13.5)	0.083
Dyslipidemia	48 (13.6)	63 (19.0)	65 (19.0)	0.090
Cardiovascular disease	33 (9.3)	34 (10.2)	32 (9.4)	0.900
Life-behavior, n (%)				
Late-night dinner	88 (24.9)	85 (25.6)	124 (36.3)	0.001
Smoking habits				
Current smoking	100 (28.3)	73 (22.0)	98 (28.7)	0.042
Past smoking	130 (36.7)	150 (45.2)	149 (43.6)	
Never	124 (35.0)	109 (32.8)	95 (27.8)	
Alcohol amount per day				
Over 60 g	17 (4.8)	21 (6.3)	20 (5.9)	0.003
40-60g	41 (11.6)	40 (12.1)	67 (19.6)	
20-40g	83 (23.5)	104 (31.3)	79 (23.1)	
0-20g	213 (60.2)	167 (50.3)	176 (51.5)	
Exercise habits				
Over 2 days/weeks	147 (41.5)	126 (38.0)	111 (32.5)	0.045
Under 2 days/weeks	207 (58.5)	206 (62.1)	231 (67.5)	
Sleeping satisfaction				
insufficient	114 (32.2)	93 (28.0)	97 (28.4)	0.406
Physical findings on admission				
Systolic blood pressure, mmHg	120 ± 15	124 ± 14	127 ± 14	0.179
Diastolic blood pressure, mmHg	76 ± 11	78 ± 10	81 ± 11	0.506
Laboratory data on admission				
Hemoglobin, mg/dL	14.6 ± 1.0	14.8 ± 1.3	15.2 ± 1.1	<0.001
AST, unit/L	21 (17, 26)	22 (18, 27)	23 (19, 29)	<0.001
ALT, unit/L	19 (14, 24)	22 (17, 31)	28 (21, 41)	<0.001
Albumin, mg/dL	4.3 ± 0.3	4.3 ± 0.3	4.3 ± 0.3	0.919
Total cholesterol, mg/dL	204 ± 36	205 ± 34	204 ± 35	0.708
Triglyceride, mg/dL	85 (64, 117)	102 (75, 143)	117 (84, 171)	<0.001
HDL-C, mg/dL	65 (56, 78)	59 (50, 70)	53 (45, 63)	<0.001
LDL-C, mg/dL	117 (97, 141)	121 (105, 142)	126 (103, 143)	0.021
FBS, mg/dL	101 ± 18	104 ± 21	107 ± 19	0.005
Creatinine, mg/dL	0.82 ± 0.10	0.83 ± 0.10	0.84 ± 0.1	0.466
Uric acid, mg/dL	5.7 ± 1.2	6.0 ± 1.2	6.4 ± 1.2	0.501
eGFR, mL/min/1.73m^2^	75.5 (69.4, 85.7)	74.0 (67.3, 83.0)	75.3 (68.1, 84.1)	0.114
Hemoglobin A1c (NGSP), %	5.6 (5.4, 5.9)	5.7 (5.5, 6.0)	5.8 (5.6, 6.1)	<0.001
Dipstick proteinuria				
Negative (-)	332 (93.8)	310 (93.4)	316 (92.4)	0.766
Trace (±)	20 (5.7)	18 (5.4)	24 (7.0)	
Mild (+)	2 (0.6)	3 (0.9)	2 (0.6)	
Moderate to heavy (2 +)	0 (0)	1 (0.3)	0 (0)	

Categorical variables are expressed as numbers (percentages) and continuous variables are shown as mean ± standard deviation or median (interquartile range), as appropriate.

BMI, body mass index; ALT, alanine aminotransferase; AST, aspartate transaminase; HDL, high-density lipoprotein; LDL, low-density lipoprotein; FBS, fasting blood sugar level; eGFR, estimated glomerular filtration rate.

**Table 1B:** Clinical characteristics of 1,099 females stratified by body mass index (BMI) levels.

Parameters	BMI<20.3 n= 383 (34.9%)	20.3 ≤BMI <23.0 n= 341 (31.0%)	23.0 ≤BMI n= 375 (34.1%)	*P* value
Age (years)	52 ± 14	55 ± 14	56 ± 14	0.761
Height (cm)	158.0 ± 5.9	156.6 ± 5.9	155.4 ± 6.4	0.194
Weight (kg)	46.8 ± 4.6	52.7 ± 4.2	63.6 ± 9.8	<0.001
BMI (kg/m^2^)	18.7 ± 1.2	21.5 ± 0.8	26.3 ± 3.5	<0.001
Waist circumference (cm)	71.8 ± 5.2	78.8 ± 5.2	90.3 ± 8.7	<0.001
Medical history, n (%)				
Hypertension	27 (7.1)	45 (13.2)	95 (25.3)	<0.001
Diabetes mellitus	8 (2.1)	16 (4.7)	34 (9.1)	<0.001
Dyslipidemia	37 (9.7)	60 (17.6)	93 (24.8)	<0.001
Cardiovascular disease	5 (1.3)	9 (2.6)	14 (3.7)	0.105
Life-behavior, n (%)				
Late-night dinner	51 (13.3)	54 (15.8)	71 (18.9)	0.108
Smoking habits				
Current smoking	31 (8.1)	13 (3.8)	29 (7.7)	0.051
Past smoking	69 (18.0)	48 (14.1)	58 (15.5)	
Never	283 (73.9)	280 (82.1)	288 (76.8)	
Alcohol amount per day				
Over 60 g	5 (1.3)	3 (0.9)	4 (1.1)	0.695
40-60g	13 (3.4)	12 (3.5)	8 (2.1)	
20-40g	32 (8.4)	37 (10.9)	43 (11.5)	
0-20g	333 (87.0)	289 (84.8)	320 (85.3)	
Exercise habits				
Over 2 days/weeks	101 (26.4)	97 (28.5)	94 (25.1)	0.589
Under 2 days/weeks	282 (73.6)	244 (71.6)	281 (74.9)	
Sleeping satisfaction				
insufficient	118 (30.8)	102 (29.9)	117 (31.2)	0.930
Physical findings on admission				
Systolic blood pressure, mmHg	113 ± 17	118 ± 18	124 ± 16	0.369
Diastolic blood pressure, mmHg	70 ± 11	72 ± 11	77 ± 11	0.918
Laboratory data on admission				
Hemoglobin, mg/dL	12.9 ± 1.1	12.9 ± 1.1	13.2 ± 1.2	0.099
AST, unit/L	19 (16, 22)	20 (16, 23)	20 (17, 24)	0.065
ALT, unit/L	15 (12, 19)	16 (13, 21)	19 (14, 26)	<0.001
Albumin, mg/dL	4.3 ± 0.3	4.2 ± 0.3	4.2 ± 0.3	0.846
Total cholesterol, mg/dL	212 ± 32	217 ± 38	213 ± 38	0.001
Triglyceride, mg/dL	65 (49, 84)	74 (57, 102)	96 (69, 130)	<0.001
HDL-C, mg/dL	81 (70, 90)	75 (64, 90)	66 (56, 76)	<0.001
LDL-C, mg/dL	115 (97, 133)	122 (103, 142)	124 (102, 146)	<0.001
FBS, mg/dL	93 ± 9	97 ± 16	102 ± 19	<0.001
Creatinine, mg/dL	0.62 ± 0.08	0.62 ± 0.08	0.61 ± 0.08	0.451
Uric acid, mg/dL	4.1 ± 0.9	4.5 ± 1.0	4.8 ± 1.0	0.337
eGFR, mL/min/1.73m^2^	77.8 (70.0, 86.3)	76.1 (69.0, 84.9)	77.2 (69.2, 86.4)	0.400
Hemoglobin A1c (NGSP), %	5.6 (5.4, 5.8)	5.7 (5.5, 5.9)	5.8 (5.6, 6.0)	<0.001
Dipstick proteinuria				
Negative (-)	368 (96.1)	335 (98.2)	358 (95.5)	0.099
Trace (±)	15 (3.9)	5 (1.5)	14 (3.7)	
Mild (+)	0 (0)	1 (0.3)	3 (0.8)	
Moderate to heavy (2 +)	0 (0)	0 (0)	0 (0)	

Categorical variables are expressed as numbers (percentages) and continuous variables are shown as mean ± standard deviation or median (interquartile range), as appropriate.

BMI, body mass index; ALT, alanine aminotransferase; AST, aspartate transaminase; HDL, high-density lipoprotein; LDL, low-density lipoprotein; FBS, fasting blood sugar level; eGFR, estimated glomerular filtration rate.

Among the participants, 297 males (28.9%) and 176 females (16.0%) took late-night dinner. In both males and females, subjects who had late-night dinner were younger. Compared with those who had dinner more than 2 hours before bedtime, those who had late-night dinner exhibited higher rates of current smokers and drinking over 60 g of ethanol (**Supplementary Tables 1A, 1B**).

To assess the association between late-night dinner and the prevalence of proteinuria, odds ratios were calculated using adjusted logistic regression models (**Tables 2A**, **2B**). Even after clinically relevant factors (model 1) and additional unhealthy behavior variables (model 2) were adjusted, late-night dinner had a significantly higher risk of proteinuria in males (adjusted odds ratios of males were as follows: model 1, 2.34 [1.42–3.87]; model 2, 2.39 [1.42–4.03], respectively). However, the association between late-night dinner and proteinuria was insignificant in females. All subjects were categorized into three subgroups stratified by BMI, as follows: males, BMI< 22.3, 22.3 ≤BMI <24.9, and BMI ≥ 24.9; and females, BMI <20.3, 20.3 ≤BMI <23.0, and BMI ≥23.0 kg/m^2^. The association between late-night dinner and the prevalence of proteinuria was evident in lower BMI males (BMI <24.9 kg/m^2^) even after adjusting for clinically relevant factors (model 1) and additional unhealthy behavior variables (model 2) (adjusted odds ratios of males: model 1, 3.10 [1.27–7.62] and 2.58 [1.06–6.29]; model 2, 3.57 [1.34–9.48] and 3.15 [1.22–8.13], respectively), whereas this association was not evident in the BMI ≥24.9 kg/m^2^ males. In females, the same association or tendency between late-night dinner and proteinuria was not shown (**Tables 3A**, **3B**).

**Table 2A:** Logistic regression analysis for the prevalence of proteinuria in male.

Male	Multivariable *Model 1		Multivariable **Model 2	
	Odds ratio (95% CI)	P-value	Odds ratio (95% CI)	P-value
Late-night dinners	2.34 (1.42-3.87)	0.001	2.39 (1.42-4.03)	0.001
Age (years)	0.99 (0.97-1.01)	0.509	0.99 (0.97-1.01)	0.629
BMI (kg/m^2^)	1.04 (0.97-1.12)	0.244	1.04 (0.97-1.11)	0.296
Medical History				
Hypertension	0.67 (0.33-1.36)	0.271	0.73 (0.36-1.51)	0.400
Diabetes mellitus	1.20 (0.54-2.70)	0.655	1.15 (0.51-2.61)	0.732
Dyslipidemia	1.27 (0.59-2.74)	0.535	1.30 (0.60-2.84)	0.504
Cardiovascular disease	0.66 (0.22-1.99)	0.464	0.71 (0.24-2.15)	0.546
Life-behavior				
Smoking habits				
Current smoking			1.40 (0.76-2.58)	0.283
Past smoking			0.72 (0.37-1.42)	0.343
Alcohol amount per day				
Over 60 g			0.43 (0.10-1.91)	0.268
40-60g			1.11 (0.54-2.26)	0.783
20-40g			1.36 (0.77-2.41)	0.293
Sleeping satisfaction insufficient			0.68 (0.38-1.21)	0.188

CI, confidence interval; BMI, body mass index.

*Adjusted for late night dinner, age (years), BMI (kg/㎡) and medical history of hypertension, diabetes mellitus, dyslipidemia and cardiovascular disease at their first visit during the study period.

**Adjusted for model 1+ smoking status (none, past, vs. current), drinking ethanol amount (0–20 g, 20–40 g, 40–60 g, vs. over 60 g) and sleeping satisfaction (sufficient, vs. insufficient) at their first visit during the study period.

**Table 2B:** Logistic regression analysis for the prevalence of proteinuria in female.

Female	Multivariable *Model 1		Multivariable **Model 2	
	Odds ratio (95% CI)	P-value	Odds ratio (95% CI)	P-value
Late-night dinners	1.47 (0.69-3.16)	0.320	1.65 (0.76-3.57)	0.205
Age (years)	0.97 (0.95-0.99)	0.050	0.97 (0.95-1.00)	0.052
BMI (kg/m^2^)	1.10 (1.02-1.17)	0.009	1.10 (1.02-1.18)	0.008
Medical history				
Hypertension	0.56 (0.15-2.10)	0.393	0.56 (0.15-2.12)	0.396
Diabetes mellitus	0.91 (0.20-4.08)	0.898	0.92 (0.20-4.17)	0.915
Dyslipidemia	1.02 (0.31-3.36)	0.977	0.90 (0.27-3.00)	0.869
Cardiovascular disease	–		–	
Life-behavior				
Smoking habits				
Current smoking			1.28 (0.37-4.43)	0.698
Past smoking			1.31 (0.55-3.08)	0.542
Alcohol amount per day				
Over 60 g			–	
40-60g			0.69 (0.09-5.33)	0.721
20-40g			0.55 (0.16-1.88)	0.341
Sleeping satisfaction insufficient			0.58 (0.26-1.29)	0.178

CI, confidence interval; BMI, body mass index.

*Adjusted for late night dinner, age (years), BMI (kg/㎡) and medical history of hypertension, diabetes mellitus, dyslipidemia and cardiovascular disease at their first visit during the study period.

**Adjusted for model 1+ smoking status (none, past, vs. current), drinking ethanol amount (0–20 g, 20–40 g, 40–60 g, vs. over 60 g) and sleeping satisfaction (sufficient, vs. insufficient) at their first visit during the study period.

**Table 3A:** Logistic regression analysis for the late night dinner and the prevalence of proteinuria in 1,028 males stratified by body mass index (BMI).

	BMI <22.3 354 (32.4%) males				22.3 ≤BMI <24.9 332 (30.4%) males				24.9 ≤ BMI 342 (31.3%) males				
	Multivariable *Model 1		Multivariable **Model 2		Multivariable *Model 1		Multivariable **Model 2		Multivariable *Model 1		Multivariable **Model 2		
	Odds ratio (95% CI)	*P* value	Odds ratio (95% CI)	*P* value	Odds ratio (95% CI)	*P* value	Odds ratio (95% CI)	*P* value	Odds ratio (95% CI)	*P* value	Odds ratio (95% CI)	*P* value
Late-night dinners	3.10 (1.27-7.62)	0.013	3.57 (1.34-9.48)	0.011	2.58 (1.06-6.29)	0.038	3.15 (1.22-8.13)	0.018	1.86 (0.82-4.23)	0.139	1.75 (0.74-4.15)	0.204
Age	0.97 (0.94-1.01)	0.114	0.97 (0.93-1.01)	0.141	0.99 (0.96-1.03)	0.751	1.00 (0.96-1.04)	0.887	1.01 (0.97-1.05)	0.597	1.01 (0.97-1.05)	0.609
Hypertension	0.54 (0.08-3.57)	0.520	0.42 (0.06-3.17)	0.401	0.83 (0.25-2.74)	0.764	0.83 (0.23-3.02)	0.781	0.61 (0.22-1.68)	0.341	0.71 (0.26-1.98)	0.515
Diabetes mellitus	0.56 (0.06-4.85)	0.599	0.45 (0.05-3.95)	0.471	1.36 (0.35-5.26)	0.654	1.28 (0.31-5.30)	0.729	1.55 (0.47-5.12)	0.468	1.33 (0.39-4.53)	0.651
Dyslipidemia	1.28 (0.21-7.84)	0.793	1.43 (0.23-9.03)	0.701	1.84 (0.51-6.71)	0.355	1.55 (0.40-6.05)	0.530	0.93 (0.28-3.06)	0.909	0.98 (0.28-3.35)	0.971
Cardiovascular disease	2.26 (0.39-13.1)	0.365	2.73 (0.43-17.3)	0.286	0.37 (0.04-3.27)	0.374	0.44 (0.05-4.11)	0.470	0.34 (0.04-2.86)	0.323	0.44 (0.05-3.74)	0.456
Smoking habits												
Current smoking			0.48 (0.15-1.50)	0.206			1.60 (0.50-5.10)	0.426			3.09 (1.04-9.18)	0.043
Past smoking			0.64 (0.19-2.18)	0.477			0.69 (0.21-2.25)	0.535			0.94 (0.27-3.28)	0.927
Alcohol amount												
Over 60 g			1.11 (0.12-10.1)	0.927			–				0.48 (0.06-4.15)	0.507
40-60g			2.55 (0.68-9.62)	0.168			1.37 (0.40-4.70)	0.621			0.47 (0.12-1.82)	0.275
20-40g			2.85 (0.99-8.18)	0.051			0.64 (0.21-1.91)	0.422			1.43 (0.55-3.67)	0.461
Sleeping												
insufficient			0.58 (0.21-1.64)	0.306			0.46 (0.14-1.46)	0.186			0.92 (0.36-2.37)	0.859

CI, confidence interval; BMI, body mass index.

*Adjusted for late night dinner, age (years), waist circumference (cm) and medical history of hypertension, diabetes mellitus, dyslipidemia and cardiovascular disease at their first visit during the study period.

**Adjusted for model 1+ smoking status (none, past, vs. current), drinking ethanol amount (0–20 g, 20–40 g, 40–60 g, vs. over 60 g) and sleeping satisfaction (sufficient, vs. insufficient) at their first visit during the study period.

**Table 3B:** Logistic regression analysis for the late-night dinner and the prevalence of proteinuria in 1,099 females stratified by body mass index (BMI).

	BMI <20.3 383 (34.9%) females				20.3 ≤BMI <23.0 341 (31.0%) females				23.0 ≤ BMI 375 (34.1%) females			
	Multivariable *Model 1		Multivariable **Model 2		Multivariable *Model 1		Multivariable **Model 2		Multivariable *Model 1		Multivariable **Model 2	
	Odds ratio (95% CI)	*P* value	Odds ratio (95% CI)	*P* value	Odds ratio (95% CI)	*P* value	Odds ratio (95% CI)	*P* value	Odds ratio (95% CI)	*P* value	Odds ratio (95% CI)	*P* value
Late-night dinners	1.96 (0.58-6.63)	0.278	2.04 (0.59-7.04)	0.257	–		–		2.24 (0.78-6.42)	0.132	2.49 (0.86-7.23)	0.094
Age	0.97 (0.93-1.01)	0.159	0.97 (0.93-1.01)	0.161	0.96 (0.90-1.03)	0.298	0.97 (0.91-1.04)	0.400	0.98 (0.94-1.02)	0.385	0.98 (0.94-1.02)	0.349
Hypertension	–		–		–		–		0.66 (0.15-2.79)	0.569	0.73 (0.17-3.15)	0.674
Diabetes mellitus	–		–		–		–		1.68 (0.34-8.34)	0.528	1.53 (0.31-7.70)	0.604
Dyslipidemia	–		–		–		–		1.52 (0.38-6.12)	0.557	1.31 (0.31-5.46)	0.712
Cardiovascular disease	–		–		–		–		–		–	
Smoking habits												
Current smoking			3.39 (0.82-14.0)	0.092			–				–	
Past smoking			1.48 (0.38-5.76)	0.576			0.65 (0.06-7.20)	0.727			1.11 (0.30-4.09)	0.875
Alcohol amount												
Over 60 g			–				–					
40-60g			–				4.90 (0.38-63.0)	0.223			–	
20-40g			0.49 (0.06-4.11)	0.513			–				0.85 (0.18-4.02)	0.836
Sleeping												
insufficient			0.56 (0.15-2.10)	0.392			0.56 (0.06-4.97)	0.599			0.70 (0.22-2.27)	0.551

CI, confidence interval; BMI, body mass index.

*Adjusted for late night dinner, age (years), waist circumference (cm) and medical history of hypertension, diabetes mellitus, dyslipidemia and cardiovascular disease at their first visit during the study period.

**Adjusted for model 1+ smoking status (none, past, vs. current), drinking ethanol amount (0–20 g, 20–40 g, 40–60 g, vs. over 60 g) and sleeping satisfaction (sufficient, vs. insufficient) at their first visit during the study period.

Waist circumference mainly reflects visceral fat accumulation (20–24). Therefore, the same analysis was performed with waist circumference. In males, 92 (27.5%), 87 (25.1%), and 118 (33.9%) took late-night dinner with waist circumference < 83.0, 83.0 ≤ waist circumference < 90.1, and 90.1 ≤ waist circumference, respectively. In females, 52 (15.1%), 62 (16.5%), and 62 (16.5%) took late-night dinner with waist circumference < 75.0, 75.0 ≤ waist circumference < 83.5, and 83.5 ≤ waist circumference, respectively. In males, 21 (6.3%), 21 (6.1%), and 27 (7.9%) showed proteinuria above ± in waist circumference < 83.0, 83.0 ≤ waist circumference < 90.1, and 90.1 ≤ waist circumference, respectively. In females, 17 (4.9%), 5 (1.3%), and 16 (4.3%) showed proteinuria above ± in waist circumference < 75.0, 75.0 ≤ waist circumference < 83.5, and 83.5 ≤ waist circumference, respectively (**Supplementary Tables 2A, 2B**).

To assess the association between late-night dinner and the prevalence of proteinuria, odds ratios were calculated using adjusted logistic regression models, including waist circumference (**Supplementary Tables 3A, 3B**). Even after clinically relevant factors (model 1) and additional unhealthy behavior variables (model 2) were adjusted, late-night dinner had a significantly higher risk of proteinuria in males (adjusted odds ratios of males were as follows: model 1, 2.37 [1.44–3.91]; model 2, 2.43 [1.44–4.09], respectively). However, the association between late-night dinner and proteinuria was insignificant in females.

The association between late-night dinner and the prevalence of proteinuria was evident in lower waist circumference males (waist circumference < 83.0 and 83.0-90.1cm), even after adjusting for clinically relevant factors (adjusted odds ratios of waist circumference < 83.0 and 83.0-90.1cm were as follows; model 1, 3.48 [1.34-9.04]; model 2, 4.41 [1.57-12.4] and model 1, 3.54 [1.43-8.74]; model 2, 3.77 [1.42-10.0], respectively), whereas this association was not evident in the waist circumference ≥ 90.1 cm males (**Supplementary Table 4A**). On the other hand, in females, the association between late-night dinner and the prevalence of proteinuria was evident in higher waist circumference (**Supplementary Table 4B**). However, in additional analysis, the association between late-night dinner and the prevalence of proteinuria was not significant in the metabolic syndrome criteria of waist circumference ≥ 90.0 cm (Data not shown). Because the females with late-night dinner had a small sample size, further research was needed to clear the association between late-night dinner and the prevalence of proteinuria in females.

## Discussion

The present study showed an additive interaction of late-night dinner and BMI on the prevalence of proteinuria across a wide range of age participants. Late-night dinner was identified as a significant predictor of proteinuria, particularly among males with lower BMI.

An association between unhealthy dietary habits and proteinuria has been reported. A cross-sectional study of 60,800 participants (38,123 men and 22,677 women) revealed that habitual breakfast skipping with late-night dinner was significantly associated with metabolic syndrome and proteinuria, even after adjusting for relevant confounders (10). In a retrospective cohort study involving 26,764 Japanese participants aged ≥ 40 years (mean age was 68 years), late-night dinner was associated with higher risks for proteinuria onset (11). This study is novel in examining the effects of gender and BMI, which were not considered in previous reports.

One of the potential mechanisms for the association between late-night dinner and the prevalence of proteinuria is that late-night dinner may cause sustained high blood glucose levels at night (25). It was reported that a decrease in insulin sensitivity and glucose oxidation in the evening resulted from higher postprandial free-fatty acid concentrations in the evening than in the morning (26). In addition, melatonin, which suppresses insulin secretion (27), has diurnal variations, with low secretion during the day and a 10-fold increase in secretion at night, which may contribute to the rise in blood glucose levels caused by late-night dinner. These mechanisms were hypothesized to induce oxidative stress and proteinuria (27).

The mechanism by which late-night dinner is associated with proteinuria in the lower BMI group remains unclear. Recent cohort studies have shown that late-night dinner is associated with an increased risk of type 2 diabetes in non-obese populations (12), suggesting that there may be different mechanisms involved compared to those observed in obese individuals. Previous studies have shown that lower BMI is linked to increased glycemic variability, mainly due to exaggerated postprandial glucose excursions. This suggests that underweight or normal-weight patients may have reduced beta-cell function compared with overweight or obese patients, leading to higher postprandial glucose levels and larger postprandial excursions in those with lower BMI (28).

Proteinuria and microalbuminuria are well known to be associated with an increased risk of cardiovascular events (1, 2). Trace proteinuria, typically defined as ± on dipstick urinalysis, roughly corresponds to microalbuminuria (16) and has been linked to metabolic syndrome, hypertension, and diabetes (17). In the present study, we focused on individuals with preserved kidney function (eGFR ≥60 mL/min/1.73 m²) and defined proteinuria as dipstick readings of ± or higher from a preventive medicine perspective. Although the inclusion of trace proteinuria may be debated, accumulating evidence indicates that even trace levels are associated with early renal stress and increased cardiovascular risk (17, 29, 30). A sensitivity analysis using proteinuria ≥1+ was also attempted, but the limited number of such cases prevented robust statistical analysis. Therefore, our findings likely reflect early renal changes detectable by trace proteinuria that may precede overt kidney damage.

In the present study, the lack of association between late-night dinner and proteinuria in females may be partly due to the influence of estrogen, which affects insulin sensitivity and suppresses the rise in blood glucose levels (31). This study did not show the association between late-night dinner and proteinuria in females because elevated blood glucose may have been suppressed. On the other hand, in males, it is speculated that aging may have decreased testosterone levels, reduced muscle mass, and insulin resistance (32). In addition, the female subgroup may have been underpowered to detect an association due to the smaller number of participants with late-night dinner and the lower prevalence of proteinuria. The wide confidence intervals and *post-hoc* power analysis indicating low statistical power suggest that the lack of a significant association in females may reflect the limited sample size rather than a true absence of relationship.

The present study has several limitations. First, the present study did not identify the more detailed frequency and content of late-night dinner. Further information is needed to determine the optimal late-night dinner frequency and content. Second, while standardized health examinations were employed, the present study was consisted of participants who spontaneously underwent health checkups at a single center in Japan; thus, the generalizability of the results needs to be verified in a multi-center study. Third, information on several important confounding variables was not available, such as the presence or absence of renin-angiotensin system inhibitor, sodium-glucose co-transporter 2 (SGLT2) inhibitor, nutritional content, shift work, and working time, which may influence the prevalence of proteinuria. In particular, information on the nutritional content and caloric load of late-night dinners and on shift work were lacking. Shift workers often have irregular meal timings and disrupted circadian rhythms, and individuals who eat late at night may have poorer overall diet quality. Therefore, residual confounding by these unmeasured factors cannot be excluded. Future studies should consider these factors to more precisely evaluate their impact on proteinuria. Fourth, regarding the research results related to females, we cannot rule out the possibility that the statistical power was insufficient due to the small number of subjects who ate dinner late at night. Fifth, the study had a small obese population (the cutoff value for the high BMI group was 24.9, with an average of 27.6 ± 2.5 kg/m^2^). Therefore, it could not clarify the relationship between late-night dinner and proteinuria in obese individuals. The relationship between obesity and proteinuria is well known, and the relationship between late-night dinner and proteinuria in obese individuals is a topic for future research.

In conclusion, the present cross-sectional study identified an association between late-night dinner and the prevalence of proteinuria in lower BMI male participants. These results suggest that proteinuria in lower BMI subjects might need to be careful about late-night dinner for males. However, the direction of causality between late-night dinner and the prevalence of proteinuria modified by BMI was unknown due to the cross-sectional study design. Even so, the results of the present study may provide clinically useful evidence indicating an association between late-night dinner and proteinuria, which could contribute to future strategies for the prevention of proteinuria.

## Data Availability

The datasets used and/or analyzed during the current study are available upon reasonable request, subject to approval by the Human Ethics Committee of Rinku General Medical Center. Requests to access the datasets should be directed to JM (muratsu@cgt.med.osaka-u.ac.jp).
