# Supplementary material for: Lower body mass index potentiates the association between late-night dinner and the prevalence of proteinuria
Source: Front Endocrinol (Lausanne). 2025 Nov 10;16:1683354. doi: 10.3389/fendo.2025.1683354 (PMC12640810; doi:10.3389/fendo.2025.1683354)
Supplement: Supplementary file 1 [file DataSheet1.pdf]

Supplementary Table 1A. Clinical characteristics of 1,028 male participants stratified on the presence of late-night dinner.

Parameters	**Late-night dinners n= 297 (28.9%)**	**Dinner more than 2 hours before bedtime n= 731 (71.1%)**	***P* value**
Age (years)	52±12	56±14	<0.001
Height (cm)	170.7±6.1	169.7±6.1	0.018
Weight (kg)	71.3±12.1	68.1±10.7	<0.001
BMI (kg/m^2)	24.4±3.8	23.6±3.2	<0.001
Waist circumference (cm)	88.2±9.8	86.5±9.1	0.008
**Medical History, n (%)**			
Hypertension	77 (25.9)	193 (26.4)	0.875
Diabetes mellitus	32 (10.8)	76 (10.4)	0.858
Dyslipidemia	39 (13.1)	137 (18.7)	0.030
Cardiovascular disease	21 (7.1)	78 (10.7)	0.076
**Life-behavior, n (%)**			
Smoking habits			
Current smoking	107 (36.0)	164 (22.4)	<0.001
Past smoking	112 (37.7)	317 (43.4)	
Never	78 (26.3)	250 (34.2)	
Alcohol amount per day			
Over 60 g	26 (8.8)	32 (4.4)	<0.001
40-60g	56 (18.9)	92 (12.6)	

20-40g	84 (28.3)	182 (24.9)	
0-20g	131 (44.1)	425 (58.1)	
Exercise habits			
Over 2 days/weeks	91 (30.6)	293 (40.1)	0.005
Under 2 days/weeks	206 (69.4)	438 (59.9)	
Sleeping satisfaction			
insufficient	121 (40.7)	183 (25.0)	<0.001
**Physical findings on admission**			
Systolic blood pressure, mmHg	125±17	124±15	0.157
Diastolic blood pressure, mmHg	80±12	78±10	0.001
**Laboratory Data on admission**			
Hemoglobin, mg/dL	15.0±1.1	14.8±1.2	0.044
AST, unit/L	22 (19, 28)	22 (18, 27)	0.072
ALT, unit/L	24 (18, 34)	22 (16, 33)	0.022
Albumin, mg/dL	4.3±0.3	4.3±0.3	0.557
Total cholesterol, mg/dL	207±35	204±34	0.161
Triglyceride, mg/dL	110 (74, 165)	99 (71, 133)	0.004
HDL-C, mg/dL	59 (50, 69)	59 (50, 71)	0.295
LDL-C, mg/dL	121 (100, 143)	121 (101, 141)	0.516
FBS, mg/dL	106±24	103±18	0.053
Creatinine, mg/dL	0.83±0.10	0.83±0.10	0.375
Uric acid, mg/dL	6.1±1.3	6.0±1.2	0.210
eGFR, mL/min/1.73m^2	76.4 (69.9, 86.8)	74.4 (67.3, 83.0)	<0.001

Hemoglobin A1c (NGSP), %	5.7 (5.4, 5.9)	5.7 (5.5, 6.0)	0.125
Dipstick proteinuria			
Negative (-)	236 (88.6)	695 (95.1)	0.001
Trace (±)	31 (10.4)	31 (4.2)	
Mild (+)	3 (1.0)	4 (0.6)	
Moderate to heavy (2 +)	0 (0.0)	1 (0.14)	

Note: Categorical variables are expressed as numbers (percentages) and continuous variables are shown as mean ± standard deviation or median (interquartile range), as appropriate.

Abbreviations: BMI, body mass index; ALT, alanine aminotransferase; AST, aspartate transaminase; HDL, high-density lipoprotein; LDL, low-density lipoprotein; FBS, fasting blood sugar level; eGFR, estimated glomerular filtration rate.

Supplementary Table1B. Clinical characteristics of 1,099 female participants stratified on the presence of late-night dinner.

Parameters	**Late-night dinners n= 176 (16.0%)**	**Dinner more than 2 hours before bedtime n= 923 (84.0%)**	***P* value**
Age (years)	49±15	55±14	<0.001
Height (cm)	157.6±6.1	156.5±6.2	0.033
Weight (kg)	56.7±11.1	53.9±9.4	<0.001
BMI (kg/㎡)	22.8±4.2	22.0±3.8	0.011
Waist circumference (cm)	81.4±11.3	80.1±10.0	0.113
**Medical History, n (%)**			
Hypertension	21 (11.9)	146 (15.8)	0.188
Diabetes mellitus	9 (5.1)	49 (5.3)	0.915
Dyslipidemia	17 (9.7)	173 (18.7)	0.003
Cardiovascular disease	3 (1.7)	25 (2.7)	0.439
**Life-behavior, n (%)**			
Smoking habits			
Current smoking	20 (11.4)	53 (5.7)	0.017
Past smoking	30 (17.1)	145 (15.7)	
Never	126 (71.6)	725 (78.6)	
Alcohol amount per day			
Over 60 g	6 (3.4)	6 (0.7)	<0.001
40-60g	8 (4.6)	25 (2.7)	

20-40g	31 (17.6)	81 (8.8)	
0-20g	131 (74.4)	811 (87.9)	
Exercise habits			
Over 2 days/weeks	41 (23.3)	251 (27.2)	0.283
Under 2 days/weeks	135 (76.7)	672 (72.8)	
Sleeping satisfaction			
insufficient	61 (34.7)	276 (29.9)	0.210
**Physical findings on admission**			
Systolic blood pressure, mmHg	116±17	119±19	0.014
Diastolic blood pressure, mmHg	72±11	73±12	0.502
**Laboratory Data on admission**			
Hemoglobin, mg/dL	12.9±1.1	13.0±1.2	0.228
AST, unit/L	18 (15, 22)	19 (17, 23)	0.004
ALT, unit/L	15 (12, 20)	16 (13, 22)	0.104
Albumin, mg/dL	4.2±0.3	4.2±0.3	0.458
Total cholesterol, mg/dL	212±33	214±36	0.629
Triglyceride, mg/dL	72 (55, 101)	77 (57, 109)	0.119
HDL-C, mg/dL	74 (62, 85)	73 (63, 86)	0.666
LDL-C, mg/dL	120 (103, 139)	119 (100, 141)	0.788
FBS, mg/dL	97±10	98±17	0.355
Creatinine, mg/dL	0.62±0.09	0.62±0.08	0.807
Uric acid, mg/dL	4.6±1.0	4.4±1.0	0.134
eGFR, mL/min/1.73m^2	79.7 (70.9, 89.9)	76.8 (69.3, 85.4)	0.031

Hemoglobin A1c (NGSP), %	5.6 (5.4, 5.8)	5.7 (5.5, 5.9)	<0.001
Dipstick proteinuria			
Negative (-)	166 (94.3)	895 (97.0)	0.004
Trace (±)	7 (4.0)	27 (2.9)	
Mild (+)	3 (1.7)	1 (0.1)	
Moderate to heavy (2 +)	0 (0.0)	0 (0.0)	

Note: Categorical variables are expressed as numbers (percentages) and continuous variables are shown as mean ± standard deviation or median (interquartile range), as appropriate.

Abbreviations: BMI, body mass index; ALT, alanine aminotransferase; AST, aspartate transaminase; HDL, high-density lipoprotein; LDL, low-density lipoprotein; FBS, fasting blood sugar level; eGFR, estimated glomerular filtration rate.

Supplementary Table 2A. Clinical characteristics of 1,028 males stratified by waist circumference.

Parameters	**Waist <83.0cm n= 334 (32.5%)**	**83.0 ≤waist <90.1 n= 346 (33.7%)**	**90.1 ≤waist n= 348 (33.8%)**	***P* value**
Age (years)	53±15	57±13	55±13	0.053
Height (cm)	169.1±5.9	169.9±6.3	170.9±6.1	0.495
Weight (kg)	59.5±6.3	67.7±6.0	79.5±10.0	<0.001
BMI (kg/m^2)	20.8±1.9	23.4±1.6	27.2±2.9	<0.001
Waist circumference (cm)	77.0±4.4	86.5±2.2	97.1±5.8	<0.001
**Medical History, n (%)**				
Hypertension	52 (15.6)	90 (26.0)	128 (36.8)	<0.001
Diabetes mellitus	30 (9.0)	31 (9.0)	47 (13.5)	0.081
Dyslipidemia	32 (9.6)	65 (18.8)	79 (22.7)	<0.001
Cardiovascular disease	26 (7.8)	38 (11.0)	35 (10.1)	0.349
**Life-behavior, n (%)**				
Late-night dinner	92 (27.5)	87 (25.1)	118 (33.9)	0.031
Smoking habits				
Current smoking	94 (28.1)	75 (21.7)	102 (29.3)	0.007
Past smoking	118 (35.3)	160 (46.2)	151 (43.4)	
Never	122 (36.5)	111 (32.1)	95 (27.3)	
Alcohol amount per day				
Over 60 g	16 (4.8)	22 (6.4)	20 (5.8)	0.001

40-60g	42 (12.6)	40 (11.6)	66 (19.0)	
20-40g	69 (20.7)	110 (31.8)	87 (25.0)	
0-20g	207 (62.0)	174 (50.3)	175 (50.3)	
Exercise habits				
Over 2 days/weeks	145 (43.4)	134 (38.7)	105 (30.2)	0.001
Under 2 days/weeks	189 (56.6)	212 (61.3)	243 (69.8)	
Sleeping satisfaction				
insufficient	113 (33.8)	88 (25.4)	103 (29.6)	0.056
**Physical findings on admission**				
Systolic blood pressure, mmHg	118±15	124±14	128±15	0.505
Diastolic blood pressure, mmHg	75±11	78±10	81±11	0.119
**Laboratory Data on admission**				
Hemoglobin, mg/dL	14.5±1.0	14.8±1.2	15.2±1.2	0.002
AST, unit/L	21 (17, 25)	22 (19, 27)	23 (19, 30)	<0.001
ALT, unit/L	19 (14, 24)	23 (17, 33)	27 (20, 40)	<0.001
Albumin, mg/dL	4.3±0.3	4.3±0.3	4.3±0.3	0.557
Total cholesterol, mg/dL	203±35	207±36	204±34	0.497
Triglyceride, mg/dL	81 (62, 112)	102 (76, 142)	119 (88, 175)	<0.001
HDL-C, mg/dL	67 (57, 79)	60 (50, 70)	53 (45, 61)	<0.001
LDL-C, mg/dL	115 (94, 138)	124 (102, 144)	125 (105, 142)	0.001
FBS, mg/dL	101±20	103±18	108±21	0.007
Creatinine, mg/dL	0.83±0.10	0.83±0.10	0.84±0.10	0.338
Uric acid, mg/dL	5.6±1.1	6.1±1.2	6.3±1.2	0.235

eGFR, mL/min/1.73m^2	76.1 (70.0, 85.8)	74.4 (67.6, 82.9)	74.1 (67.4, 84.1)	0.045
Hemoglobin A1c (NGSP), %	5.6 (5.4, 5.8)	5.7 (5.5, 6.0)	5.8 (5.6, 6.1)	<0.001
Dipstick proteinuria				
Negative (-)	313 (93.7)	325 (93.9)	320 (92.0)	0.739
Trace (±)	19 (5.7)	18 (5.2)	25 (7.2)	
Mild (+)	2 (0.6)	3 (0.9)	2 (0.6)	
Moderate to heavy (2 +)	0 (0)	0 (0)	1 (0.3)	

Note: Categorical variables are expressed as numbers (percentages) and continuous variables are shown as mean ± standard deviation or median (interquartile range), as appropriate.

Abbreviations: BMI, body mass index; ALT, alanine aminotransferase; AST, aspartate transaminase; HDL, high-density lipoprotein; LDL, low-density lipoprotein; FBS, fasting blood sugar level; eGFR, estimated glomerular filtration rate.

Supplementary Table 2B. Clinical characteristics of 1,099 females stratified by waist circumference.

Parameters	**Waist <75.0cm n= 345 (31.4%)**	**75.0 ≤waist <83.5 n= 377 (34.3%)**	**83.5 ≤waist n= 377 (34.3%)**	***P* value**
Age (years)	50±14	55±14	57±13	0.137
Height (cm)	156.8±5.9	157.1±6.3	156.2±6.3	0.265
Weight (kg)	46.9±4.7	52.4±5.0	63.2±10.0	<0.001
BMI (kg/m²)	19.1±1.6	21.2±1.7	25.9±3.8	<0.001
Waist circumference (cm)	69.6±3.5	78.9±2.4	91.4±7.5	<0.001
**Medical History, n (%)**				
Hypertension	21 (6.1)	52 (13.8)	93 (25.4)	<0.001
Diabetes mellitus	11 (3.2)	15 (4.0)	32 (8.5)	0.002
Dyslipidemia	29 (8.4)	62 (16.5)	99 (26.3)	<0.001
Cardiovascular disease	5 (1.5)	8 (2.1)	15 (4.0)	0.080
**Life-behavior, n (%)**				
Late-night dinner	52 (15.1)	62 (16.5)	62 (16.5)	0.847
Smoking habits				
Current smoking	24 (7.0)	16 (4.2)	33 (8.8)	0.139
Past smoking	56 (16.2)	57 (15.1)	62 (16.5)	
Never	265 (76.8)	304 (80.0)	282 (74.8)	
Alcohol amount per day				
Over 60 g	3 (0.9)	5 (1.3)	4 (1.1)	0.504

40-60g	13 (3.8)	12 (3.2)	8 (2.1)	
20-40g	27 (7.8)	40 (10.6)	45 (11.9)	
0-20g	302 (87.5)	320 (84.9)	320 (84.9)	
Exercise habits				
Over 2 days/weeks	78 (22.6)	115 (30.5)	99 (26.3)	0.055
Under 2 days/weeks	267 (77.4)	262 (69.5)	278 (73.7)	
Sleeping satisfaction				
insufficient	105 (30.4)	109 (28.9)	123 (32.6)	0.539
**Physical findings on admission**				
Systolic blood pressure, mmHg	113±16	117±18	125±17	0.115
Diastolic blood pressure, mmHg	69±10	73±11	76±11	0.237
**Laboratory Data on admission**				
Hemoglobin, mg/dL	12.8±1.2	13.0±1.1	13.3±1.2	0.436
AST, unit/L	18 (16, 22)	20 (17, 23)	20 (17, 24)	<0.001
ALT, unit/L	14 (12, 19)	16 (13, 20)	19 (14, 26)	<0.001
Albumin, mg/dL	4.3±0.3	4.2±0.2	4.2±0.3	0.010
Total cholesterol, mg/dL	209±34	216±35	216±39	0.016
Triglyceride, mg/dL	61 (47, 82)	76 (58, 101)	100 (71, 138)	<0.001
HDL-C, mg/dL	81 (71, 92)	76 (65, 85)	65 (56, 77)	<0.001
LDL-C, mg/dL	114 (96, 132)	121 (103, 141)	125 (105, 148)	<0.001
FBS, mg/dL	93±10	96±11	103±22	<0.001
Creatinine, mg/dL	0.62±0.08	0.63±0.08	0.61±0.08	0.323
Uric acid, mg/dL	4.0±0.9	4.5±0.9	4.8±1.0	0.015

eGFR, mL/min/1.73m^2	78.9 (71.0, 89.5)	76.0 (68.4, 84.2)	76.5 (69.7, 85.9)	0.005
Hemoglobin A1c (NGSP), %	5.6 (5.4, 5.8)	5.7 (5.5, 5.9)	5.8 (5.6, 6.1)	<0.001
Dipstick proteinuria				
Negative (-)	328 (95.1)	372 (98.7)	361 (95.8)	0.014
Trace (±)	17 (4.9)	4 (1.1)	13 (3.5)	
Mild (+)	0 (0)	1 (0.3)	3 (0.8)	
Moderate to heavy (2 +)	0 (0)	0 (0)	0 (0)	

Note: Categorical variables are expressed as numbers (percentages) and continuous variables are shown as mean ± standard deviation or median (interquartile range), as appropriate.

Abbreviations: BMI, body mass index; ALT, alanine aminotransferase; AST, aspartate transaminase; HDL, high-density lipoprotein; LDL, low-density lipoprotein; FBS, fasting blood sugar level; eGFR, estimated glomerular filtration rate.

Supplementary Table 3A. Logistic regression analysis for the prevalence of proteinuria in males.

Male	Multivariable *Model 1		Multivariable **Model 2	
	**Odds ratio (95% CI)**	**p-value**	**Odds ratio (95% CI)**	**p-value**
**Late-night dinners**	2.37 (1.44-3.91)	0.001	2.43 (1.44-4.09)	0.001
**Age (years)**	0.99 (0.97-1.01)	0.405	0.99 (0.97-1.01)	0.530
**Waist circumference (cm)**	1.01 (0.98-1.04)	0.527	1.01 (0.98-1.03)	0.659
**Medical History**				
**Hypertension**	0.70 (0.34-1.41)	0.345	0.76 (0.37-1.57)	0.460
**Diabetes mellitus**	1.23 (0.55-2.76)	0.613	1.18 (0.52-2.66)	0.689
**Dyslipidemia**	1.28 (0.59-2.75)	0.536	1.31 (0.60-2.85)	0.502
**Cardiovascular disease**	0.67 (0.22-2.01)	0.474	0.72 (0.24-2.16)	0.553
**Life-behavior**				
**Smoking habits**				
**Current smoking**			1.40 (0.76-2.58)	0.282
**Past smoking**			0.72 (0.37-1.42)	0.349
**Alcohol amount per day**				
**Over 60 g**			0.42 (0.10-1.88)	0.258
**40-60g**			1.12 (0.55-2.29)	0.761
**20-40g**			1.36 (0.76-2.41)	0.297
**Sleeping satisfaction**				
**insufficient**			0.68 (0.38-1.21)	0.188

Abbreviations: CI, confidence interval.

* Adjusted for late night dinner, age (years), waist circumference (cm) and medical history of hypertension, diabetes mellitus, dyslipidemia and cardiovascular disease at their first visit during the study period.

** Adjusted for model 1+ smoking status (none, past, vs. current), drinking ethanol amount (0-20 g, 20-40 g, 40-60 g, vs. over 60 g) and sleeping satisfaction (sufficient, vs. insufficient) at their first visit during the study period.

Supplementary Table 3B. Logistic regression analysis for the prevalence of proteinuria in females.

	Multivariable *Model 1		Multivariable **Model 2	
	Odds ratio (95% CI)	p-value	Odds ratio (95% CI)	p-value
**Late-night dinners**	1.57 (0.73-3.36)	0.249	1.74 (0.80-3.76)	0.161
**Age (years)**	0.97 (0.95-0.99)	0.04	0.97 (0.95-0.99)	0.041
**Waist circumference (cm)**	1.01 (0.98-1.04)	0.513	1.01 (0.98-1.04)	0.458
**Medical History**				
**Hypertension**	0.68 (0.18-2.52)	0.566	0.70 (0.19-2.60)	0.590
**Diabetes mellitus**	1.05 (0.23-4.69)	0.951	1.05 (0.23-4.72)	0.950
**Dyslipidemia**	1.05 (0.32-3.48)	0.934	0.95 (0.28-3.15)	0.928
**Life-behavior**				
**Smoking habits**				
**Current smoking**			1.29 (0.37-4.44)	0.690
**Past smoking**			1.28 (0.54-3.00)	0.575
**Alcohol amount per day**				
**40-60g**			0.63 (0.08-4.90)	0.661
**20-40g**			0.57 (0.17-1.94)	0.369
**Sleeping satisfaction**				
**insufficient**			0.58 (0.26-1.28)	0.178

Abbreviations: CI, confidence interval.

* Adjusted for late night dinner, age (years), waist circumference (cm) and medical history of hypertension, diabetes mellitus, dyslipidemia and cardiovascular disease at their first visit during the study period.

** Adjusted for model 1+ smoking status (none, past, vs. current), drinking ethanol amount(0-20 g, 20-40 g, 40-60 g, vs. over 60 g) and sleeping satisfaction (sufficient, vs. insufficient) at their first visit during the study period.

Supplementary Table 4A. Logistic regression analysis for the late-night dinner and the prevalence of proteinuria in 1,028 males stratified by waist circumference.

	**Waist circumference < 83.0cm 334 (32.5%) males**				**83.0 ≤ Waist circumference < 90.1cm 346 (33.7%) males**				**90.1 cm ≤ Waist circumference 348 (33.8%) males**			
	Multivariable *Model 1		Multivariable **Model 2		Multivariable *Model 1		Multivariable **Model 2		Multivariable *Model 1		Multivariable **Model 2	
	Odds ratio (95% CI)	*P* value	Odds ratio (95% CI)	*P* value	Odds ratio (95% CI)	*P* value	Odds ratio (95% CI)	*P* value	Odds ratio (95% CI)	*P* value	Odds ratio (95% CI)	*P* value
Late-night dinners	3.48 (1.34-9.04)	0.011	4.41 (1.57-12.4)	0.005	3.54 (1.43-8.74)	0.006	3.77 (1.42-10.0)	0.008	1.44 (0.65-3.22)	0.372	1.32 (0.57-3.04)	0.517
Age	0.98 (0.94-1.01)	0.210	0.98 (0.94-1.02)	0.253	1.00 (0.96-1.04)	0.924	1.00 (0.96-1.05)	0.867	0.99 (0.96-1.03)	0.866	0.99 (0.96-1.04)	0.915
Hypertension	0.35 (0.05-2.32)	0.277	0.42 (0.06-2.79)	0.366	0.67 (0.18-2.57)	0.561	0.63 (0.15-2.74)	0.539	0.728 (0.28-1.88)	0.513	0.87 (0.33-2.28)	0.780
Diabetes mellitus	0.91 (0.18-4.53)	0.906	1.05 (0.24-4.50)	0.949	1.27 (0.25-6.37)	0.773	0.72 (0.08-6.11)	0.760	1.42 (0.44-4.59)	0.557	1.28 (0.38-4.24)	0.692
Dyslipidemia	6.46 (1.14-36.7)	0.035	7.48 (1.29-43.5)	0.025	0.82 (0.20-3.41)	0.785	0.64 (0.12-3.38)	0.596	0.92 (0.31-2.72)	0.874	0.88 (0.29-2.70)	0.828
Cardiovascular disease	0.85 (0.09-8.36)	0.886	1.82 (0.27-12.4)	0.542	1.15 (0.20-6.48)	0.876	0.63 (0.06-6.13)	0.690	0.33 (0.04-2.67)	0.300	0.39 (0.05-3.23)	0.386
Smoking habits												
Current smoking			1.02 (0.35-2.92)	0.974			0.66 (0.17-2.53)	0.542			2.62 (0.94-7.28)	0.065
Past smoking			0.35 (0.09-1.40)	0.138			0.90 (0.28-2.87)	0.864			0.80 (0.24-2.60)	0.705
Alcohol amount												
Over 60 g			-				0.59 (0.06-5.81)	0.650			0.46 (0.05-3.85)	0.470
40-60g			1.88 (0.56-6.35)	0.307			1.15 (0.28-4.79)	0.843			0.64 (0.19-2.15)	0.470
20-40g			1.37 (0.46-4.09)	0.574			1.03 (0.34-3.08)	0.960			1.46 (0.59-3.64)	0.415
Sleeping insufficient			0.81 (0.30-2.19)	0.673			0.41 (0.11-1.49)	0.175			0.84 (0.34-2.12)	0.718

Abbreviations: CI, confidence interval.

* Adjusted for late night dinner, age (years), waist circumference (cm) and medical history of hypertension, diabetes mellitus, dyslipidemia and cardiovascular disease at their first visit during the study period.

** Adjusted for model 1+ smoking status (none, past, vs. current), drinking ethanol amount (0-20 g, 20-40 g, 40-60 g, vs. over 60 g) and sleeping satisfaction (sufficient, vs. insufficient) at their first visit during the study period.

Supplementary Table 4B. Logistic regression analysis for the late-night dinner and the prevalence of proteinuria in 1,099 females stratified by waist circumference.

	**Waist circumference < 75.0cm 345 (31.4%) females**				**75.0 ≤ Waist circumference < 83.5cm 377 (34.3%) females**				**83.5 cm ≤ Waist circumference 377 (34.3%) females**			
	Multivariable *Model 1		Multivariable **Model 2		Multivariable *Model 1		Multivariable **Model 2		Multivariable *Model 1		Multivariable **Model 2	
	Odds ratio (95% CI)	*P* value	Odds ratio (95% CI)	*P* value	Odds ratio (95% CI)	*P* value	Odds ratio (95% CI)	*P* value	Odds ratio (95% CI)	*P* value	Odds ratio (95% CI)	*P* value
Late-night dinners	1.78 (0.54-5.90)	0.347	1.87 (0.54-6.54)	0.324	-		-		3.08 (1.05-9.02)	0.040	3.53 (1.17-10.68)	0.025
Age	0.99 (0.95-1.03)	0.489	0.99 (0.95-1.03)	0.494	0.95 (0.88-1.03)	0.188	0.96 (0.88-1.03)	0.266	0.96 (0.92-1.00)	0.076	0.96 (0.92-1.00)	0.048
Hypertension	-		-		-		-		0.98 (0.22-4.42)	0.980	1.28 (0.27-6.07)	0.752
Diabetes mellitus	-		-		-		-		2.48 (0.48-12.90)	0.281	2.36 (0.43-12.80)	0.320
Dyslipidemia	1.38 (0.15-12.52)	0.775	1.51 (0.16-14.26)	0.721	-		-		1.04 (0.22-4.98)	0.961	0.78 (0.15-4.06)	0.772
Cardiovascular disease	-		-		-		-		-		-	
Smoking habits												
Current smoking			3.76 (0.91-15.61)	0.068			-				-	
Past smoking			1.26 (0.33-4.77)	0.730			0.64 (0.05-9.01)	0.744			0.88 (0.23-3.40)	
Alcohol amount												
Over 60 g			-				-				-	
40-60g			-				8.54 (0.53-136.89)	0.130			-	
20-40g			0.55 (0.07-4.52)	0.581			-				0.76 (0.15-3.77)	0.739
Sleeping insufficient			0.45 (0.12-1.66)	0.231			0.57 (0.06-5.68)	0.629			0.63 (0.19-2.12)	0.460

Abbreviations: CI, confidence interval.

* Adjusted for late night dinner, age (years), waist circumference (cm) and medical history of hypertension, diabetes mellitus, dyslipidemia and cardiovascular disease at their first visit during the study period.

** Adjusted for model 1+ smoking status (none, past, vs. current), drinking ethanol amount (0-20 g, 20-40 g, 40-60 g, vs. over 60 g) and sleeping satisfaction (sufficient, vs. insufficient) at their first visit during the study period.
